# Comparison of dental surface of canine teeth in dogs after crown preparation using CBCT-based surface reconstruction or conventional dental impression materials

**DOI:** 10.3389/fvets.2025.1722332

**Published:** 2025-12-04

**Authors:** Benjamin Metje, Jan Schreyer, Matthias Lüpke, Sebastian Meller, Jan R. S. Klasen, Jerzy Pawel Gawor

**Affiliations:** 1 University of Veterinary Medicine Hannover, Foundation, Hannover, Germany; 2 Tierärztliche Gemeinschaftspraxis am Kaßberg, Dr. Plümer & Dr. Schreyer, Chemnitz, Germany; 3 Tierklinik Germersheim GmbH, Germersheim, Germany

**Keywords:** cone beam computed tomography, CBCT, digital impression, accuracy, prosthodontics, stone model

## Abstract

**Objective:**

This cadaver study evaluated accuracy of Cone Beam Computed Tomography (CBCT)-derived surface models in reproducing crown-prepared canine teeth and compared them with conventional impressions/plaster models as the current gold standard in veterinary prosthodontics.

**Methods:**

Forty canine teeth underwent crown preparation. Conventional impressions with plaster model fabrication and CBCT scans at two resolutions (n = 20 0.12 mm, n = 20 0.09 mm) were obtained. Models and original teeth were digitized by precise surface scanning. Surface distance from plaster models and CBCT datasets was compared with the original teeth (reference). Mean deviations were calculated and compared statistically.

**Results:**

Plaster models showed deviations of 9.3–56.4 μm. High-resolution CBCT scans yielded 21.3–36.4 μm; low-resolution scans 30.6–514.5 μm, with one outlier excluded. Within both cohorts, CBCT and plaster model deviations did not differ significantly; however, the two CBCT protocols differed significantly (*p* = 0.0058), with higher resolution producing lower deviations.

**Conclusion:**

Both CBCT protocols produced deviations comparable to plaster models in this in-vitro single-tooth setup. Higher-resolution CBCT improved accuracy, but all CBCT datasets remained within clinically acceptable ranges. CBCT may represent a feasible alternative for digital impressions in veterinary prosthodontics, though radiation exposure and clinical applicability must be considered.

## 1 Introduction

Historically, impressions were first described in the 18th century to reproduce the intraoral anatomy in humans and were regarded as a technique of exceptionally high accuracy and reproducibility (1–3). Over the last decades, on the human side, more digital impression methods like intraoral scanners (IOS) were investigated and proved comparable accuracy with further evolution of the technique (4–6). The benefits of a digital workflow in dentistry are obvious: practitioners can avoid different sources of errors in connection with the process of conventional impression technique, save on materials, and have direct feedback on the quality of their reproduction of the situation (6–9). In addition, the dental laboratories increasingly employ Computer-Aided Design/Computer-Aided Manufacturing (CAD/CAM) technology, which allows the implementation of digital impressions for a standardized and seamless workflow.

Indications for obtaining partial or full mouth impressions in veterinary dentistry are not as common as in human dentistry, but they do exist. For example, in cases that require a prosthodontic crown, the dental laboratory has to be provided with a detailed replication of the intraoral anatomy to allow for a high-quality crown production (10, 11). Unfortunately, literature only shows little evidence for the application of digital technology in veterinary dentistry. In 2019, CAD/CAM was shown to be a useful tool for manufacturing accurate prosthodontic crowns for dogs as veterinary patients, highlighting the possibility of employing a procedure that has long since become part of everyday dentistry in the human field (12). And while one case report described the use of an IOS in a dog with malocclusion, a recent study showed that the accuracy of an IOS used to capture the surface of crown-prepared canine teeth in dogs is within a clinically acceptable range (13, 14). Thus, an IOS could be used in single teeth of dogs to obtain a digital impression; however, the costs for these devices are high and therefore not always sustainable. Considering the rarity of indications in veterinary practice, the scanner might appear to be a financially questionable investment for many in our profession.

On the other hand, diagnostic imaging in veterinary dentistry has further developed over the past decade, as well. While dental radiographs were proven to be an irreplaceable tool in a standard clinical setting for many reasons, advanced methods of 3D imaging have also been proven to exceed the diagnostic value of 2D imaging in certain setups and, therefore, have been implemented in a growing number of practices and clinics (15, 16). Considering their substantially lower costs compared to conventional Computed Tomography, smaller CBCT units may represent an attractive investment for practitioners, particularly with an interest in dentistry. Consequently, their availability has markedly increased in recent years among veterinary hospitals and practices. Although CBCT is primarily employed as a diagnostic modality, its growing accessibility as well as studies on the human site raise the question of whether the volumetric datasets it generates may also be repurposed for use in prosthodontic applications (17). If CBCT-derived datasets demonstrate sufficient accuracy, they could potentially serve as a basis for generating digital impressions for the dental laboratory. This would represent a significant advantage, as many veterinary practices already have access to CBCT scanners. Therefore, investigating the feasibility of CBCT-based digital impressions in veterinary patients appears to be of considerable clinical and practical relevance to the authors. The aim of this study was to compare the accuracy of CBCT-derived surface reconstructions with conventional stone models after crown preparation of canine teeth in dogs.

## 2 Materials and methods

As a first step for this study, a total of five cadavers were obtained from the clinical service of the Department of Small Animal Medicine and Surgery of the University of Veterinary Medicine Hannover, Germany. Three of the dogs obtained were euthanized due to reasons unrelated to this study. The other two dogs were deceased upon arrival at the clinic. All of them were frozen directly after euthanasia/arrival. In addition, five frozen heads of dogs of unknown sex, breed and age were included, for a total of 10 heads (n = 40 canine teeth). The whole group was then defrosted for 2 days at room temperature before conducting the study.

The goal was to mimic crown fractures as a possible indication for later prosthodontic crowning. Therefore, all canine teeth underwent a reduction of the clinical crown by cutting the tip with a diamond bur (round end taper FG, iM3 dental, Duleek, Ireland) at random height to mimic the variety of clinical situations presented in veterinary practices. A standard crown preparation was performed, designing a chamfer margin by using different diamond burs (round end taper FG and round 6/8 FG, iM3 dental, Duleek, Ireland) as used in a clinical setting. With a focus not to damage the prepared area of the teeth, all of them were surgically extracted afterwards and underwent a disinfection protocol (Peroxy AG+, Stern Weber, Imola, Italy).

As a next step after complete drying, detailed impressions of each tooth were obtained by a fifth-year resident of the European Veterinary Dental College (BM), using hydrophilic addition polymerization type silicon rubber impression material with mixing base & catalyst (iM3 Soft Impression Putty plus iM3 2-part Impression Material, iM3 dental, Duleek, Ireland) in a custom-made tray. Initially, a preliminary imprint was taken with a high-viscosity silicone putty. Base and catalyst were used in identical shares (precisely 3.0 g each, determined on a fine-scale balance) and mixed manually, in exact accordance with the manufacturer’s instructions. Following the polymerization of the putty, space within the imprint was gained by removing a thin layer of approximately 2 mm of material from the inner part of the preliminary impression for application of the low-viscosity material to capture fine details of the tooth surface. The two components of the light-body were mixed within the self-mixing tips provided by the manufacturer before the phase was applied to the teeth and the preliminary imprint. In contrast to the monophase strategy, the two-step protocol allows for elevated hydraulic pressure during insertion, thereby enhancing the distribution of material into recesses of limited accessibility. The outcome is a more detailed imprint of the clinical scenario. Each impression underwent visual inspection (BM, trained dental technician) to confirm a satisfactory reproduction of the preparation and the absence of flaws like voids or defects.

After a minimum of 30 min to allow for viscoelastic recovery of the set impression material, conventional stone models were fabricated by a dental laboratory using hard plaster (Girostone pastel, Amann Girrbach, Mäder, Austria) according to the manufacturer’s detailed instructions. The surfaces of the dried stone models, as well as those of the teeth, were subsequently scanned using a CAD/CAM high-resolution blue-light scanner (ceramill map 600, Amann Girrbach, Mäder, Austria). Stereolithography (STL) files of all 40 tooth surfaces and all 40 plaster model surfaces were acquired with an accuracy of 0.004 mm. The specimens were listed in the random order in which they were processed. To divide them into two cohorts, the first 20 teeth on the list and their corresponding scans were assigned to Group A, while the following 20 were assigned to Group B. No additional criteria were applied, and the allocation was without any systematic selection.

Afterwards, all original teeth have been scanned by a CBCT-scanner (NewTom 7G, NewTom, Cefla S.C., Imola, Italy) within 24 h. Therefore, the teeth were arranged in groups of four and placed into play-dough for the scan. Conventional cannulas were added to the material, serving as an indicator and ensuring correct identification within the scans afterwards. The settings for all scans were as follows:

- Slice thickness: 0.12 mm
- Dosage: 28 mA/120 kV
- Preset: Best quality—boosted dose
- Field of view: 6 × 6 cm.

The first cohort of 20 teeth (Group A) was scanned with

- Pixel spacing: 0.12/0.12 mm
- 520/522 rows/columns.

While the second cohort of 20 teeth (Group B) was scanned with

- Pixel spacing: 0.09/0.09 mm
- 674/672 rows/columns.

All scans were processed using the same software (NNT Version 16.3.1, NewTom, Cefla S.C., Imola, Italy). For each group of four specimens, a single DICOM file was exported from the CBCT scanner and imported into Amira 3D Pro (Thermo Fisher Scientific, Waltham, Massachusetts, USA), where the individual teeth were segmented and converted into surface models. In contrast, the STL files of the teeth and the stone models—generated directly by the CAD/CAM surface scanner—already represented finalized surface datasets and required no additional preprocessing. Thus, Amira 3D Pro received three inputs per tooth: the CBCT-based DICOM data for segmentation and surface creation, and the two STL surface models for direct use in the subsequent analysis steps. As CBCT inherently produces volumetric image data, they were converted into a surface to allow the use of the “surface alignment” function of the software, later. Therefore, the original CT datasets were pre-processed using the function “median filter” of Amira 3D Pro. This non-linear filter reduces noise by replacing each voxel intensity with the median of its local neighbourhood, thereby suppressing outliers while preserving sharp structural boundaries such as air-dentin interfaces. Thereafter, the outer contour of the tooth was segmented by applying an automatic thresholding procedure based on Otsu’s method, which determines an optimal threshold value by minimizing intra-class intensity variance. This step provided an objective and reproducible delineation of the tooth surface from the surrounding background. The root canal was digitally closed using the “fill hole” tool of the software, which then enabled the application of the “generate surface” function to convert the voxel-based object into a 3D surface. To ensure consistency, the region of the pulp opening at the crown tip was manually outlined and digitally closed using the corresponding function in Amira 3D Pro across all three produced surfaces per tooth. Additionally, a rectangular region of interest was defined around the prepared crown area and roughly aligned with the individual crown margin for all 40 teeth.

The surfaces of the three models were aligned (overlapped) for each canine tooth using manual alignment initially, followed by a detailed alignment using the “align surfaces” function within the region of interest. A relative root mean square threshold of 0.001 was established as the convergence criterion, with the surface scan of the original tooth serving as the reference for this alignment. Upon achieving alignment per the defined root mean square criterion, the “surface distance” function was implemented, again referencing the original tooth scan and facilitating a point-to-nearest-point comparison. The software subsequently generated mean deviation values in millimeters for each tooth (which were converted to micrometers manually to facilitate clarity), with comparisons conducted separately between the original tooth surface scan and both the stone model surface scan and the surface derived from the CBCT data of each group. Hence, the database to be evaluated included one value for the comparison of the stone model scan to the original tooth and one value for the comparison of the CBCT scan per tooth and among both cohorts.

### 2.1 Statistical analysis

All datasets were first assessed for normality using the Shapiro–Wilk test, which indicated non-parametric distribution for all variables.

For each cohort (Group A and Group B), the mean deviation values obtained from the stone model scan and the CBCT-derived surface of the same tooth were compared using the Wilcoxon Signed-Rank Test (two-tailed), as these data represent paired measurements referring to identical anatomical specimens.

To evaluate the effect of the two different CBCT scanning protocols applied in Group A and Group B, an unpaired comparison of the CBCT deviation values between both groups was performed using a two-tailed Mann–Whitney U Test, as the values originate from different teeth.

Statistical significance was defined as *p* < 0.05 for all tests.

## 3 Results

The signalment of the included dogs reflected one neutered female, two intact males, and two neutered male dogs. The sample comprised two mixed-breed dogs and single representatives of the Bearded Collie, Galgo Español, and German Shepherd breeds. The mean age of those cadavers was calculated at 8.85 years (8 years and 10.2 months), with a range from 5 years and 8 months to 17 years and 5 months, while the median age was 6.3 years (6 years and 4 months). All dogs weighed over 20 kg (median: 26.8 kg; mean: 26.68 kg; range 21.0–32.4 kg). In addition, heads from five large dogs of unknown sex, age, and breed were also used.

**Figure 1:**
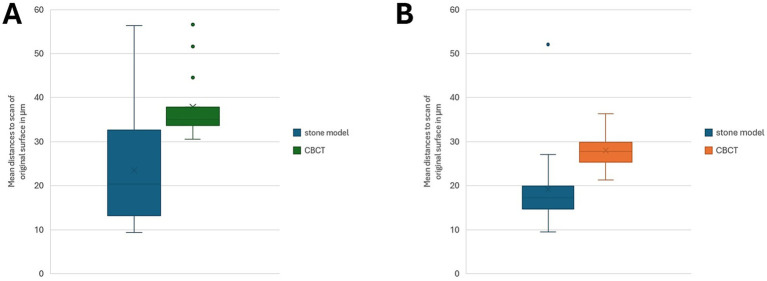
**(A,B)** Box-and-whisker plots illustrating mean distances (in μm) between the original tooth surface scans and the corresponding reconstruction modalities for Group A (**A**, *n* = 19) and Group B (**B**, *n* = 20). No statistically significant differences within the groups were revealed using the Wilcoxon Signed-Rank Test (*Group A*: W-value 279, Z-value −1.327, *p* = 0.184; *Group B*: W-value 371, Z-value −0.52, *p* = 0.603).

The reported distances for Group A were between 9.34 and 56.4 μm (mean: 24.12 μm, SD 11.58 μm) for the stone models and between 30.6 and 514.5 μm (mean: 61.67 μm, SD 106.82 μm) for the CBCT scan (Figure 1A). For Group B the variations for the stone models were between 9.5 and 52.1 μm (mean: 19.17 μm, SD 8.94 μm), while for the CBCT scans, distances between 21.31 and 36.35 μm (mean: 27.97 μm, SD 3.6 μm) were calculated (Figure 1B).

One CBCT-scan of group A showed outlining high deviations (514.495 μm), and the specimen was excluded from the statistical analysis. Therefore, the corrected mean of the stone model distances was 23.47 μm (SD 11.53 μm) and for the CBCT distances 37.92 μm (SD 7.49 μm).

The comparison of mean deviations between the stone model and CBCT-derived surfaces within Group A (n = 19 teeth) using the Wilcoxon Signed-Rank Test revealed no statistically significant difference. The test yielded a W-value of 279, and a Z-value of −1.327 (*p* = 0.184). Similarly, for Group B, the Wilcoxon Signed-Rank Test comparing the stone model and CBCT surfaces also showed no significant difference (W = 371, Z = −0.52, *p* = 0.603). In other words, the results indicate that within each cohort, CBCT-derived deviations were not significantly different from those of the conventional stone models, relative to the original tooth surface scans.

**Figure 2:**
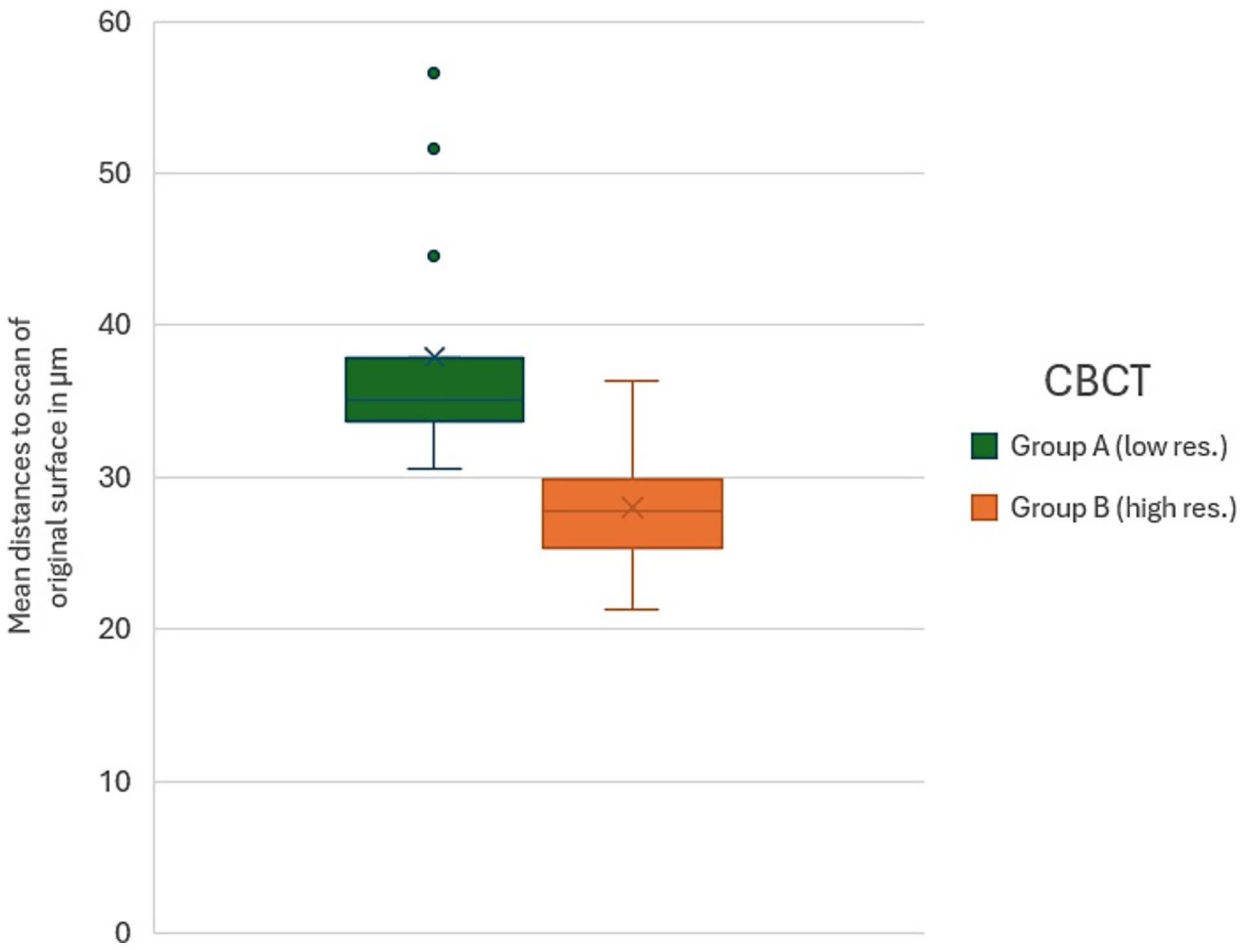
Box-and-whisker plot comparing mean distance values (in μm) of the CBCT-derived surface models to the reference surface (original tooth) between Group A (low-resolution protocol, 0.12 mm voxels) and Group B (high-resolution protocol, 0.09 mm voxels). Mann–Whitney U testing revealed a statistically significant difference between the two CBCT protocols (*p* = 0.00578), indicating improved accuracy of digital imprints under the higher-resolution settings.

In contrast, the comparison of CBCT-derived surface deviations between Group A and Group B (Figure 2) using the Mann–Whitney U Test revealed a statistically significant difference between the two scanning protocols (Z = 2.76013, *p* = 0.00578). This suggests that the CBCT scanning settings applied in Group B produced systematically lower deviations compared to Group A, indicating a measurable effect of the scanning parameters on the accuracy of CBCT-derived surface models.

## 4 Discussion

This study evaluated the accuracy of CBCT in reproducing the surface geometry of crown-prepared canine teeth, comparing the resulting datasets to both the original tooth surfaces and conventional plaster model scans, representing the current gold standard. One CBCT scan in Group A, showing extremely high deviations (514.5 μm), was excluded from the analysis. The Wilcoxon Signed-Rank tests revealed that, within each cohort, deviations measured from CBCT scans were not significantly different from those of the stone models, indicating that, in general, CBCT provides comparable surface accuracy. However, the Mann–Whitney U test demonstrated a significant difference between the two CBCT scanning protocols, with the higher-resolution settings in Group B producing systematically lower deviations than the lower-resolution protocol in Group A. Specifically, Group A exhibited higher mean deviations overall, whereas Group B consistently achieved lower deviations, closely approximating those obtained from the stone models, which remain the gold standard for dental impressions. These findings suggest that while CBCT is a feasible method for digital impressions, the scanning protocol substantially influences the accuracy of the resulting surface models, with higher-resolution settings offering improved fidelity.

The voxel volumes of the two CBCT protocols differed notably: the low-resolution scans had isotropic voxels of 0.12 × 0.12 × 0.12 mm (volume = 0.001728 mm^3^), whereas the high-resolution scans had slightly anisotropic voxels of 0.09 × 0.09 × 0.12 mm (volume = 0.000972 mm^3^), representing a reduction of approximately 44% in voxel volume. The reduced voxel size was associated with the improved measurement accuracy observed, while the uniform acquisition protocol across samples maintained internal comparability. These findings align with other studies from the human field, highlighting the influence of voxel size on the accuracy of CBCT reconstructions (17, 18). A recent study showed that voxel size might not be a significant factor in accuracy, but highlighted the importance of the used software—a variable that the present study did not attempt to evaluate (19).

The deviations observed in both CBCT groups fell within the range generally considered clinically acceptable in human dentistry, where marginal discrepancies below 120 μm and internal spaces of 20–100 μm are frequently cited (20). This suggests that CBCT scanning may represent a viable alternative for generating digital surface models under the conditions described in veterinary prosthodontics. However, unlike conventional impression-based workflows or other digital methods like IOS, CBCT reconstructions come with additional practical and methodological considerations, which warrant closer examination. The technical foundation of any radiologically derived surface reconstruction largely defines its utility in prosthodontic applications. Parameters such as voxel size, field of view, exposure settings, reconstruction algorithms, and detector properties interact to set the ultimate limits of trueness and precision (17, 19, 21). Multiple investigations have demonstrated that decreasing voxel size typically enhances the geometric fidelity of CBCT reconstructions; however, this improvement is accompanied by larger datasets, longer reconstruction times, and often higher sensitivity to other sources of error. This trade-off is of particular importance for precise surface reconstruction as needed in the prosthodontic field.

Translating in-vitro findings into the clinical environment introduces the complicating factor of motion artefacts. What is technically feasible under scientific conditions with extracted teeth can be seriously compromised by spontaneous head movements due to respiration or other minor shifts during in-vivo scanning. Motion has been consistently documented as a major determinant of image degradation in CBCT datasets among different indications, and although motion-correction algorithms are under development, their current effectiveness is inconsistent and highly dependent on device type and motion pattern (22, 23). For veterinary applications, where sedation or general anesthesia is usually required, scan duration becomes an important determinant of both image quality and patient safety: longer scans increase anesthetic risk and, in addition, raise the likelihood of motion-related artefacts, underscoring the importance of balancing resolution with practicality. A recently published systematic review on the time efficiency between conventional impressions and IOS scans in different settings on the human site found no clear advantage of one method (24). On the other hand, according to the manufacturer’s information, scanning time even in higher resolutions still should not exceed the time needed for standard impressions or IOS scans, which in the case of veterinary dentistry, would also have to be performed under general anesthesia. Therefore, CBCT still appears to be a legitimate option to acquire adequate reproduction of the intraoral situation when needed.

Modern CBCT systems address the trade-off between image quality and radiation exposure by implementing features such as pulsed exposure, adaptive dose modulation, small focal spots, and isotropic voxel geometries. Many platforms additionally offer adjustable voxel sizes and automatic exposure control, which provide operators with the flexibility to choose between low-dose, fast acquisitions and longer, high-resolution protocols. While these technical options enhance versatility, they do not in themselves guarantee clinical applicability; protocol selection must still be tailored to the specific indication, anesthesia considerations, and the anticipated diagnostic or prosthetic benefit (25).

Compared to conventional impression techniques, CBCT-based approaches eliminate several transformation steps—impression material to plaster cast to scanned surface—each of which has been shown to potentially introduce cumulative distortions in some cases (3, 26). This simplification can reduce sources of error and accelerate workflow. However, CBCT inevitably entails an additional burden of radiation. Following the ALARA principle (“as low as reasonably achievable”), any radiographic exposure must be justified by added diagnostic value (27). In a purely prosthodontic context, this justification appears to be weak; CBCT as a mere surrogate for impressions can therefore be considered debatable unless accompanied by other clinically relevant radiological indications or integrated into broader diagnostic assessments. In this case, it comes with the benefit of saving any additional time for acquiring impressions at all, as the patient will be scanned anyway.

The clinical usability of the observed deviations warrants nuanced interpretation. While numerical differences on a level of micrometers may or may not reach statistical significance *in vitro*, they do not necessarily equal clinical relevance. In human dentistry, marginal gaps up to a certain level, as outlined above, are often considered acceptable, and studies repeatedly demonstrate that restorations with minor discrepancies remain functionally successful (20). For veterinary dentistry, however, these thresholds cannot be simply transferred: bite forces, periodontal response, and practical aspects of prosthesis design differ substantially, and only outcome-oriented studies assessing fit, function, and longevity in animals will ultimately determine whether the deviations measured in CBCT-derived models are clinically meaningful (28). To the knowledge of the authors, evidence on these aspects is missing and could be a valuable topic for further research.

One distinctive advantage of CBCT, rarely matched by IOS or conventional impressions, lies in its comprehensive volumetric representation of the dentition. Provided that scanning can be performed without an endotracheal tube obstructing occlusion, the resulting dataset can capture not only detailed surface reconstructions but also an integrated view of adjacent teeth and their occlusal relationships—without requiring additional time. This contrasts with conventional impressions or intraoral scanner workflows, which necessitate separate bite registrations or extra scans to obtain this information.

From a methodological perspective, future work should ideally take the next step beyond reporting mean deviations to include sensitivity analyses (e.g., with and without outliers), systematic evaluation of different voxel and reconstruction protocols, simulation of motion artifacts under in-vivo conditions, and—most importantly—validation against clinical endpoints such as marginal fit, occlusal accuracy, and restoration survival. A comprehensive assessment like that could help determine whether the observed numerical differences, even in the absence of statistical significance in this *in vitro* study, might have practical relevance in a clinical setting of prosthodontic procedures.

In conclusion, CBCT-based surface reconstructions—particularly when performed in higher resolutions used in this study—can achieve accuracy comparable to IOS and conventional impressions in an in-vitro, single tooth setup. Their clinical adoption, however, must weigh radiation dose and procedural feasibility against the potential diagnostic and workflow benefits. If the comprehensive information provided by CBCT, including occlusal and skeletal context, is clinically required, then its use may be justified as a multifunctional tool, allowing for significant time savings. For a simple replacement for conventional impressions, however, the accuracy of CBCT should be further studied, especially in a clinical setting.

## Data Availability

The raw data supporting the conclusions of this article will be made available by the authors, without undue reservation.
